# A Peculiar Disease in a Young Woman Wanting to Get Pregnant

**DOI:** 10.4274/tjh.galenos.2021.2021.0191

**Published:** 2021-12-07

**Authors:** Tülin Tiraje Celkan, Şeyma Fenercioğlu, Ayşe Gonca Kaçar

**Affiliations:** 1 İstinye University Faculty of Medicine, Department of Pediatric Hematology-Oncology, İstanbul, Turkey; 2 İstanbul IVF Center, İstanbul, Turkey; 3 İstanbul University-Cerrahpaşa, Cerrahpaşa Faculty of Medicine, Department of Pediatric Hematology-Oncology, İstanbul, Turkey

**Keywords:** Ligneous membranes, Ligneous cervicitis, Plasminogen, Rare disease

## To the Editor,

Plasminogen has an important role in intravascular and extravascular fibrinolysis [1,2,3]. Severe hypoplasminogenemia is associated with ligneous conjunctivitis, but ligneous lesions can also occur in many different mucosal membranes like the cervix and endometrium [4,5]. Despite numerous clinical evaluations, biopsies, and laboratory tests, these kinds of diagnoses remained elusive for many years [6]. By presenting this case, we want to increase the awareness of this peculiar disease for its proper diagnosis.

**Figures 1 and 2:**
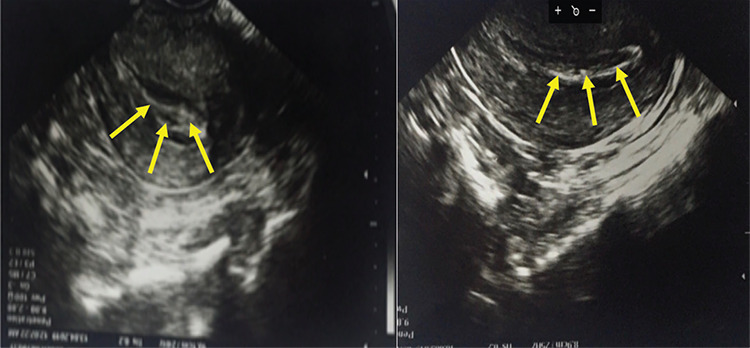
Colposcopic and ultrasound examination of the uterus revealed a woody membranous lesion that covered the inner part of the uterine cavity; a regular cavity view was achieved after treatment.

A 32-year-old nulliparous woman was referred to our clinic. Her history revealed persistent conjunctivitis that started when she was 10 years old. She had married 5 years ago and the couple wanted to have a baby. Despite extensive and detailed investigations, no definitive reason for lack of conception was found other than right fallopian tube occlusion. After serological and genetic tests were done in our clinic, she was diagnosed with plasminogen deficiency (*Plg *gene mutation Lys38Glu and plasminogen level of <0.01 mg/dL, while normal levels are 0.06-0.25 mg/dL). The patient’s family health history revealed that she was the only symptomatic member of the family. She was referred to a gynecologist due to infertility and cervicitis. Colposcopic and ultrasound examination (Figures 1 and 2) of the uterus revealed a woody membranous lesion that covered the inner part of the uterine cavity. The pathological evaluation of membranes from endocervical curetting showed woody fibrin accumulation, patchy ulceration, and polymorphonuclear leukocyte exudate [5]. Her menstrual cycles were normal and she had no problems during intercourse [6]. As plasma-derived plasminogen concentrate (NCT02312180) was not available in Turkey, fresh frozen plasma (FFP) was given both intravenously and directly into the uterine cavity in an off-label way. Our clinical practice with FFP and alteplase, a recombinant human tissue-type plasminogen activator (rTPA) administered by bronchoscopic method, was published recently [7]. Therefore, a similar regimen was utilized for this case. FFP was given intravenously on alternate days and 50 mL of FFP with 5 mg of rTPA was also administered into the uterine cavity and held there for 4 hours. When a regular cavity view was achieved after FFP and rTPA treatment (Figures 1 and 2), frozen embryo transfer was performed three times. Unfortunately, pregnancy could not be achieved despite the pre-preparation of the genital tract for embryonal transfer with FFP and rTPA. Pantanowitz et al. [8] also mentioned infertility in women with plasminogen deficiency in their work. Our local treatment response was successful in the bronchial tree, but not in the genital tract. In our opinion, the failure of the treatment can be attributed to plasmin due to its role in the degradation of the follicular wall during ovulation [8]. Impaired ovarian function may be the cause of infertility besides the woody membranous lesions in the uterine cavity.

Awareness about plasminogen deficiency and its symptoms are essential for early diagnosis and treatment of this challenging disease [9]. Currently, plasma-derived plasminogen concentrate is reported to resolve all complications, except infertility [6]. However, 25% of female patients have only genital tract ligneous infiltration of the cervix and uterus accompanying infertility. Therefore, new therapy modalities, like our therapy, should be developed and tried in this group of patients to reduce morbidity and mortality.
